# The effect on survival of continuing chemotherapy to near death

**DOI:** 10.1186/1472-684X-10-14

**Published:** 2011-09-21

**Authors:** Akiko M Saito, Mary Beth Landrum, Bridget A Neville, John Z Ayanian, Craig C Earle

**Affiliations:** 1 Laboratory of Clinical, Epidemiological and Health Services Research, Clinical Research Center, National Hospital Organization Nagoya Medical Center, Aichi, Japan; 2 Department of Health Care Policy, Harvard Medical School, Boston, MA, USA; 3 Division of Population Sciences, Department of Medical Oncology, Dana-Farber Cancer Institute, Boston, MA, USA; 4 Division of General Medicine, Brigham and Women's Hospital, Boston, MA, USA; 5 Health Services Research Program, Cancer Care Ontario and the Ontario Institute for Cancer Research, Toronto, ON, Canada

## Abstract

**Background:**

Overuse of anti-cancer therapy is an important quality-of-care issue. An aggressive approach to treatment can have negative effects on quality of life and cost, but its effect on survival is not well-defined.

**Methods:**

Using the Surveillance, Epidemiology, and End Results-Medicare database, we identified 7,879 Medicare-enrolled patients aged 65 or older who died after having survived at least 3 months after diagnosis of advanced non-small cell lung cancer (NSCLC) between 1991 and 1999. We used Cox proportional hazards regression analysis, propensity scores, and instrumental variable analysis (IVA) to compare survival among patients who never received chemotherapy (n = 4,345), those who received standard chemotherapy but not within two weeks prior to death (n = 3,235), and those who were still receiving chemotherapy within 14 days of death (n = 299). Geographic variation in the application of chemotherapy was used as the instrument for IVA.

**Results:**

Receipt of chemotherapy was associated with a 2-month improvement in overall survival. However, based on three different statistical approaches, no additional survival benefit was evident from continuing chemotherapy within 14 days of death. Moreover, patients receiving chemotherapy near the end of life were much less likely to enter hospice (81% versus 51% with no chemotherapy and 52% with standard chemotherapy, P < 0.001), or were more likely to be admitted within only 3 days of death.

**Conclusions:**

Continuing chemotherapy for advanced NSCLC until very near death is associated with a decreased likelihood of receiving hospice care but not prolonged survival. Oncologists should strive to discontinue chemotherapy as death approaches and encourage patients to enroll in hospice for better end-of-life palliative care.

## Background

The paucity of information regarding cancer prognosis, treatment options, and the possible consequences of treatment make it difficult for terminally ill patients to make appropriate end-of-life treatment choices,[1-4] despite a growing awareness of the importance of high quality end-of-life care. We have previously shown that the aggressive use of chemotherapy in patients who are close to death has been increasing over time [5]. In fact, although an aggressive approach to treatment during the last week of life is linked to psychological and physical distress for advanced cancer patients,[6,7] little information is available about the clinical effects of such treatment [8]. A systematic review of the literature from the past two decades found that patients would choose chemotherapy near death for much smaller expected benefits in outcome than would health care providers,[4] indicating a skepticism on the part of physicians for this kind of care. Other studies have also shown that chemotherapy is used near death irrespective of the cancer's responsiveness to therapy [9,10]. Such treatment has been associated with potentially negative effects, including higher numbers of emergency room (ER) visits, hospitalizations, and admissions to the intensive care unit (ICU), and less hospice service [5,11]. These results beg the question of whether aggressive care leads to improved outcomes. To address this issue, we evaluated the effect of an aggressive approach to care, defined as continuation of chemotherapy within two weeks of death, on survival in a cohort of patients with metastatic non-small cell lung cancer.

## Methods

### Definition of standard chemotherapy and aggressive-approach chemotherapy

The receipt of chemotherapy for each patient was identified from billing claims [12]. For the purpose of this study, we defined 'no chemotherapy' patients as those who never received chemotherapy after their cancer diagnosis, 'standard chemotherapy' recipients as those who did receive chemotherapy for their cancer but not during the 14 days prior to death, and 'aggressive-approach chemotherapy' recipients as those who were still receiving chemotherapy within 14 days of death [13].

### Data sources and identification of the study cohort

The study was approved by the Dana-Farber/Partners Cancer Care Institutional Review Board. The data for this study were extracted from the linked Surveillance, Epidemiology, and End Results (SEER)-Medicare database, compiled by the National Cancer Institute. Eleven tumor registries participated in the SEER program during the period of study. Approximately 97% of all the cancer cases that occur in the regions encompassed by the registries are captured,[14] covering a representative sample of approximately 14% of the United States population [15,16]. For each patient, SEER registries collect data on age, gender, race/ethnicity, cancer site, stage, histology, date of cancer diagnosis, and date of death. Data from the 2000 Census, such as the median and per capita income and wealth, have been merged with the registry data. Claims for inpatient and outpatient care, physician and laboratory services, and hospice care were retrieved from the Medicare database.

The potentially eligible study cohort included 15,391 patients who were 65 years or older, were diagnosed with metastatic non-small cell lung cancer, and died of their disease between 1991 and 1999. This cohort did not include patients who were enrolled in Medicare for end-stage renal failure or disability instead of older age, and those whose cancer diagnoses were detected from autopsy or death certificates. Patients who did not have continuous Medicare enrolment (Part A and Part B) or who were enrolled in a health maintenance organization (HMO) at any time in the year prior to death were also excluded. When estimating survival among three treatment groups in the potentially eligible study cohort, aggressive-approach chemotherapy recipients died very quickly compared to standard chemotherapy recipients (P < 0.05, data not shown), suggesting the possibility of high proportion of patients with rapid disease progression in the aggressive-approach chemotherapy group or a negative effect of such care. To eliminate the first case, we excluded patients who died within three months from their diagnosis (n = 7,512). This is consistent with the eligibility criteria for most clinical protocols that examine the effects of chemotherapy, which usually require patients to have at least a three-month prognosis. Thus, a total of 7,879 patients were included in the final cohort. The excluded cohorts had a tendency not to receive any chemotherapy compared with the study cohort who survived at least 3 months (15% versus (vs.) 45%), while the proportion receiving chemotherapy near the end of life were the same between the two groups (4% vs. 4%), suggesting the choice of aggressive-approach chemotherapy was not influenced by patients' prognoses. We also confirmed that the inclusion of the excluded cohort did not change the results in sensitivity analyses. These findings support the fact that the study cohort was valid and aggressive-approach chemotherapy recipients were not just those with poor prognosis.

### Statistical analysis

#### 1) Definition and classification of explanatory variables

Patients were categorized into three treatment groups: no chemotherapy, standard chemotherapy, or an aggressive approach to chemotherapy. Control variables included patient age at diagnosis, gender, race/ethnicity (non-Hispanic white, non-Hispanic black, Hispanic, or other), geographic region (Northeast, South, Midwest, or West), urban residence (densely-settled area with more than 2,500 residences-yes or no), whether they were treated at a teaching hospital at any time between cancer diagnosis and death (yes or no), and year of death. A Charlson comorbidity index (0, 1, or 2 or more) was calculated by using the algorithm described elsewhere [17-19]. Socioeconomic quintiles were developed following the method described by Bach et al. [20]; Only eighty-six patients for whom no income data were available were grouped in the lowest quintile of socioeconomic status, and exclusion of their data did not change the results in sensitivity analyses. Based on billing information, patients were divided into three groups according to the length of hospice care (none, three or fewer days, or four or more days).

#### 2) Analytical approach

##### Unadjusted comparison

Descriptive statistical analyses to assess baseline sociodemographic and disease characteristics among patients in the three treatment groups were performed using Chi-square tests for categorical variables and Wilcoxon rank-sum tests for continuous variables, with the Kruskal-Wallis test used for overall comparisons. Overall survival (OS) was calculated using the Kaplan-Meier method,[21] and a log-rank test [22] was used for group comparisons.

##### Adjusted comparison-1: multiple Cox regression analysis

We used a Cox proportional hazards regression model to compare relative risks and 95% confidence intervals (95%CIs) for mortality in the three treatment groups, unadjusted and adjusted for 9 key factors: patient age, gender, the Charlson comorbidity index, race/ethnicity, geographic region, urban residence, socioeconomic status, teaching hospital, and year of death. Any variables that were significantly associated with the receipt of chemotherapy in the univariate analyses were considered for inclusion in the model. Significant variables associated with survival were identified through stepwise selection, and interaction terms that revealed significant effect modification (i.e. the interaction term with treatment group was significant) were further investigated.

##### Adjusted comparison-2: propensity score analysis

Propensity score (PS) approaches have been proposed as a less parametric alternative when there are large observed differences between treatment groups. The PS, introduced by Rosenbaum and Rubin in 1983,[23] is the conditional probability of assignment to a certain treatment procedure, given sociodemographic and disease characteristics. PS methods thus permit control for all observed confounding factors that might influence both choice of treatment and outcome using a single composite measure, without requiring specification of the relationships between the control variables and outcome, which differs from a Cox proportional hazards regression model.

We first calculated the propensity for receiving chemotherapy with a logistic regression model, which includes all the control variables. We examined the overlap in PS among patients who received chemotherapy and those who did not to ensure the suitability of comparing treatment outcome between the two groups. We then stratified the sample of patients into five propensity strata. Within each stratum, we estimated survival separately using a Cox proportional hazards regression model. A multivariable Cox regression adjustment using PS as a continuous variable was also performed.

The same process was repeated for patients who ever received chemotherapy: the propensity for undergoing an aggressive approach to chemotherapy was calculated, patients were divided into five propensity strata, and survival was estimated using both a Cox proportional hazards regression model within each stratum in which confounding factors were substantially reduced [23,24] and a multivariable Cox regression adjustment using PS as a continuous variable.

##### Adjusted comparison-3: instrumental variable analysis

The two preceding statistical tools do not directly consider unobserved factors that may be unrelated to observed variables, but may nevertheless affect treatment choices and/or their outcomes. The instrumental variable analysis (IVA) method, first developed in the field of econometrics [25] and applied to the field of health care research since the mid-1990s,[12,26-29] permits the consideration of not only observed factors, but also unobserved factors that might influence outcomes.

The instrumental variable (IV) in an IVA should independently influence patients' or physicians' treatment choice, but should not be associated with outcomes. On the basis of findings from several published studies,[30,31] we used treatment rates within Health Care Service Areas (HCSAs),[32] a classification of geographic areas based on observed referral patterns for tertiary care, and stratified by the availability of health care resources, as the IV. To confirm the assumption of this analysis, we included the IVs in a Cox proportional hazards regression model to determine that it would not predict survival independently. Also, we confirmed that sociodemographic and disease characteristics across IVs are more similar than when we compared those characteristics among the three treatment groups.

First, we calculated the numbers of elderly patients receiving chemotherapy for advanced lung cancer in each HCSA. HCSAs with five or fewer patients treated with or without chemotherapy were excluded. The remaining HCSAs were divided into quintiles based on the proportion of patients receiving chemotherapy. We then calculated the IV estimates for the "marginal patient population," which is defined as patients who would receive chemotherapy if they lived in a HCSA with a high chemotherapy utilization rate but not if they lived in a HCSA with low chemotherapy utilization rate [33]. A non-parametric two-stage least squares model was used to predict treatment in the first stage based on IV quintiles. These models also controlled for patient age, race/ethnicity, gender, socioeconomic status, Charlson comorbidity index, and the year of death. Among those who ever received chemotherapy, we took the same approach from the perspective of more and less aggressive-approach chemotherapy utilization and estimated the clinical effect of receiving chemotherapy within 14 days of death for the marginal patient population.

All tests were two-tailed, and P-values less than 0.05 were considered significant. All analyses were performed using SAS software, version 9.1 (SAS Institute, Cary, NC).

## Results

### Patient characteristics

Table 1 summarizes the characteristics of the study population. Of the 7,879 patients, 3,534 patients (44.9%) had ever received chemotherapy, and 299 (8.5%) of those patients were still receiving chemotherapy within 14 days of death.

**Table 1:** Characteristics of 7,879 patients with metastatic non-small cell lung cancer by receipt of chemotherapy

Variables		No chemotherapy (n = 4,345)	Standard chemotherapy (n = 3,235)	Aggressive -approach chemotherapy (n = 299)	P-value		
					
					3 groups	No chemo-therapy vs. Chemo- therapy*	Standard chemotherapy vs. Aggressive -approach chemotherapy
Age at diagnosis, years	Median [IQR]	73.0 [69.0-78.0]	71.0 [68.0-74.0]	70.0 [67.0-74.0]	< 0.001	< 0.001	0.36
Gender, n (%)	Female	1,938 (44.6)	1,291 (39.9)	108 (36.1)	< 0.001	< 0.001	0.20
	Male	2,407 (55.4)	1,944 (60.1)	191 (63.9)			
Charlson comorbidity index, n (%)	0	3,123 (71.9)	2,401 (74.2)	226 (75.6)	< 0.001	< 0.001	0.06
	1	773 (17.8)	576 (17.8)	60 (20.1)			
	2 ≤	449 (10.3)	258 (8.0)	13 (4.4)			
Race/Ethnicity, n (%)	non-Hispanic white	3,551 (81.7)	2,711 (83.8)	254 (85.0)	0.01	0.01	0.22
	non-Hispanic black	425 (9.8)	248 (7.7)	21 (7.0)			
	Hispanic or other	369 (8.5)	276 (8.5)	24 (8.0)			
Region of tumor registries, n (%)	Northeast	672 (15.5)	555 (17.2)	51 (17.1)	< 0.01	< 0.01	0.06
	South	228 (5.3)	194 (6.0)	28 (9.4)			
	Midwest	1,426 (32.8)	1,101 (34.0)	86 (28.8)			
	West	2019 (46.5)	1385 (42.8)	134 (44.8)			
Urban residence, n (%)	Yes	3,928 (90.4)	2,973 (91.9)	284 (95.0)	< 0.01	< 0.01	0.06
	No	417 (9.6)	262 (8.1)	15 (5.0)			
Socioeconomic status, n (%)	Highest quintile	763 (17.6)	682 (21.1)	71 (23.8)	< 0.001	< 0.001	0.85
	Fourth quintile	793 (18.3)	673 (20.8)	63 (21.1)			
	Third quintile	866 (19.9)	688 (21.3)	59 (19.7)			
	Second quintile	849 (19.5)	635 (19.6)	56 (18.7)			
	Lowest quintile	1,074 (24.7)	557 (17.2)	50 (16.7)			
Median income	Median [IQR]	38,154 [29-152]	41,205 [32-757]	43,564 [32-179]	< 0.001	< 0.001	0.28
Teaching hospital, n (%)	Yes	2,110 (48.6)	1,868 (57.7)	163 (54.5)	< 0.001	< 0.001	0.28
	No	2,235 (51.4)	1,367 (42.3)	136 (45.5)			
Year of death, n (%)	1991	365 (8.4)	206 (6.4)	18 (6.0)	< 0.001	< 0.001	0.02
	1992	576 (13.3)	299 (9.2)	22 (7.4)			
	1993	567 (13.1)	340 (10.5)	15 (5.0)			
	1994	581 (13.4)	339 (10.5)	33 (11.0)			
	1995	518 (11.9)	372 (11.5)	33 (11.0)			
	1996	490 (11.3)	424 (13.1)	32 (10.7)			
	1997	442 (10.2)	413 (12.8)	46 (15.4)			
	1998	421 (9.7)	421 (13.0)	52 (17.4)			
	1999	385 (8.9)	421 (13.0)	48 (16.1)			
Death in the hospital, n (%)	Yes	774 (17.8)	812 (25.1)	152 (50.8)	< 0.001	< 0.001	< 0.001
	No	3,571 (82.2)	2,423 (74.9)	147 (49.2)			
ICU admission within 1 month of death, n (%)	Yes	187 (4.3)	213 (6.6)	37 (12.4)	< 0.001	< 0.001	< 0.001
	No	4,158 (95.7)	3,022 (93.4)	262 (87.6)			
More than 1 ER visit within 1 month of death, n (%)	Yes	870 (20.0)	857 (26.5)	104 (34.8)	< 0.001	< 0.001	< 0.01
	No	3,475 (80.0)	2,378 (73.5)	195 (65.2)			
Hospice admission, n (%)	None	2,208 (50.8)	1,668 (51.6)	241 (80.6)	< 0.001	< 0.001	< 0.001
	Three or fewer days	156 (3.6)	198 (6.1)	25 (8.4)			
	Four or more days	1,981 (45.6)	1,369 (42.3)	33 (11.0)			

Abbreviation: vs., versus; IQR, interquartile range; ICU, intensive care unit; ER, emergency room.

*Chemotherapy includes standard chemotherapy or aggressive-approach chemotherapy

Several factors differed between patients who received chemotherapy and those who did not. Recipients of chemotherapy, whether standard or aggressive-approach chemotherapy, were younger, more likely to be male, and less likely to have comorbidity compared with non-recipients. The chemotherapy group had more non-Hispanic white patients and those who were more likely to reside in the Northeast or South, in urban areas, be admitted to teaching hospitals, have higher socioeconomic status, and to have died in more recent years compared with the no chemotherapy group. Among patients who ever received chemotherapy, recipients of chemotherapy near death had less comorbidity and tended to have died in more recent years, were more likely to reside in the South, were less likely to reside in the Midwest, and more often resided in urban areas compared to standard chemotherapy recipients.

### Unadjusted outcomes

**Figure 1:**
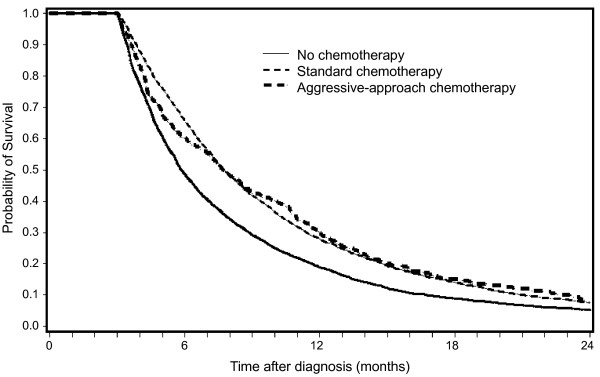
**Unadjusted survival among for metastatic non-small cell lung cancer patients by receipt of chemotherapy**. Three lines indicate patients who never received chemotherapy (solid line), those who received standard chemotherapy (dashed line), and those who received an aggressive chemotherapy approach continued to within 14 days of death (thick dashed line).

Deaths in the hospital, ER visits and ICU admissions were more common in patients who received standard chemotherapy compared to patients who did not receive chemotherapy, and each of these outcomes occurred even more frequently in recipients of chemotherapy near death (Table 1). Over 40% of patients in the group without chemotherapy or those in the standard chemotherapy group had hospice stays of four or more days, compared with only 11% of those in the aggressive-approach chemotherapy group; in fact, over 80% of those receiving chemotherapy near death received no hospice care (Table 1). The unadjusted survival rates among the three groups by receipt of chemotherapy are shown in Figure 1. The median survival of patients who received no chemotherapy, standard chemotherapy, and aggressive-approach chemotherapy were 6 months, 8 months, and 8 months, respectively. Survival of these three groups at 1 year was 18.6% for the group with no chemotherapy, 27.2% for the standard chemotherapy group, and 28.6% for the aggressive-approach chemotherapy group, respectively (3 groups, P < 0.001; no chemotherapy vs. any chemotherapy, P < 0.001; standard chemotherapy vs. aggressive-approach chemotherapy, P = 0.83).

### Adjusted outcomes

#### 1) Results from Cox model

In the Cox regression analysis, adjusted hazard ratios (HR) of patients with standard chemotherapy or chemotherapy near death were significantly lower compared to patients who received no chemotherapy, overlapping confidence intervals between recipients of standard and aggressive-approach chemotherapy approaches (standard chemotherapy, HR = 0.80, 95%CI 0.76, 0.83, P < 0.001; aggressive-approach chemotherapy, HR = 0.82, 95%CI 0.72, 0.92, P < 0.001, Table 2). When making standard chemotherapy the reference group (data not shown), no chemotherapy was associated with worse survival (HR = 1.26, 95%CI 1.20, 1.32, P < 0.001) and chemotherapy near the end of life did not have any impact on survival compared to standard chemotherapy (HR = 1.03, 95%CI 0.91, 1.16, P = 0.66). Older age at diagnosis, male gender, worse comorbidity index, and residence in the West were associated with worse survival. Race/ethnicity, urban residence, socioeconomic status, and treatment in teaching hospitals were not related to survival. Significantly improved survival was evident in 1998 and 1999 relative to 1991 (Table 2).

**Table 2:** Factors significantly associated with survival in multiple Cox regression analysis

Variables		HR	(95%CI)	P-value
Receipt of chemotherapy	No chemotherapy	1.00	-	-
	Standard chemotherapy	0.80	(0.76, 0.83)	< 0.001
	Aggressive-approach chemotherapy	0.82	(0.72, 0.92)	< 0.001
Age at diagnosis	65	1.00	-	-
	Each increasing year	1.01	(1.00, 1.01)	< 0.001
Gender	Female	1.00	-	-
	Male	1.09	(1.05, 1.14)	< 0.001
Charlson comorbidity index	0	1.00	-	-
	1	1.16	(1.09, 1.23)	< 0.001
	2 ≤	1.28	(1.18, 1.38)	< 0.001
Region of tumor registries	Northeast	1.00	-	-
	South	-	-	n.s.
	Midwest	-	-	n.s.
	West	1.08	(1.03, 1.13)	< 0.001
Socioeconomic status	Lowest quintile	1.00	-	-
	Each increasing quintile	-	-	n.s.
Teaching hospital	No	1.00	-	-
	Yes	-	-	n.s.
Year of death	1991	1.00	-	-
	1992	1.11	(1.04, 1.19)	< 0.01
	1993	-	-	n.s.
	1994	-	-	n.s.
	1995	-	-	n.s.
	1996	-	-	n.s.
	1997	-	-	n.s.
	1998	0.91	(0.84, 0.97)	< 0.01
	1999	0.89	(0.82, 0.96)	< 0.01

Abbreviation: HR, hazard ratio; CI, confidence interval; n.s., not significant.

NOTE: Stepwise selection was used. Race/ethnicity, and living in urban region were not found to be significant predictors of survival in univariate analysis (P-value > 0.20).

#### 2) Results from propensity score analyses

Care in a teaching hospital was more likely to be provided to chemotherapy recipients compared to non-recipients, whereas among patients who ever received chemotherapy, those in teaching hospitals were less likely to receive chemotherapy near death. Prognostic factors were well balanced across the propensity strata. There was considerable overlap in the PSs of chemotherapy recipients compared to non-recipients. Mean PS to receive chemotherapy was 0.50 (95%CI 0.23, 0.77) in the chemotherapy group and was 0.41 in the group with no chemotherapy (95%CI 0.12, 0.70). Standard chemotherapy recipients had a mean PS to receive aggressive-approach chemotherapy of 0.08 (95%CI 0.02, 0.15), while those with aggressive-approach chemotherapy had a mean PS of 0.10 (95%CI 0.03, 0.16).

The Cox regression model using the PS is shown in Table 3. The Cox regression adjustment using the PS as a continuous variable showed that the HR for chemotherapy recipients was 0.76 (95%CI 0.71, 0.82, P < 0.001), and those stratified by the PS quintiles found that it ranged from 0.73 to 0.85 across propensity strata (all P < 0.01). Of those who received any chemotherapy (Table 4), the HR for aggressive-approach chemotherapy recipients was 1.21 (95%CI 1.00, 1.48, P = 0.05) from the Cox regression adjustment using the PS as a continuous variable. There appeared to be a trend of decreasing hazards with a greater propensity to receive chemotherapy near death, ranging from 1.32 to 0.85, and the interaction between receipt of an aggressive approach to chemotherapy and propensity quintiles was marginally significant (P = 0.07).

**Table 3:** Propensity score analysis results 1: Survival impact across quintiles based on the propensity to receive chemotherapy among patients with metastatic non-small cell lung cancer, who survived at least 3 months after their cancer diagnosis (n = 7,879)

Analysis	Variables	HR	(95%CI)	P-value
Stratified analysis				
Lowest quintile of PS*	No chemotherapy	1.00	-	-
	Chemotherapy	0.82	(0.73, 0.93)	< 0.01
Second quintile of PS	No chemotherapy	1.00	-	-
	Chemotherapy	0.77	(0.69, 0.85)	< 0.001
Third quintile of PS	No chemotherapy	1.00	-	-
	Chemotherapy	0.81	(0.74, 0.90)	< 0.001
Fourth quintile of PS	No chemotherapy	1.00	-	-
	Chemotherapy	0.85	(0.77, 0.93)	< 0.01
Highest quintile of PS^†^	No chemotherapy	1.00	-	-
	Chemotherapy	0.73	(0.66, 0.81)	< 0.001
Multiple Cox regression adjustment using the PS	No chemotherapy	1.00	-	-
	Chemotherapy	0.76	(0.71, 0.82)	< 0.001

Abbreviation: HR, hazard ratio; CI, confidence interval; PS, propensity score.

* Patients are least likely to receive "chemotherapy."

† Patients are most likely to receive "chemotherapy."

**Table 4:** Propensity score analysis results 2: Survival impact across quintiles based on the propensity to receive aggressive-approach chemotherapy within 14 days of death among chemotherapy recipients with metastatic non-small cell lung cancer, who survived at least 3 months after their cancer diagnosis (n = 3,534)

Analysis	Variables	HR	(95%CI)	P-value
Stratified analysis				
Lowest quintile of PS*	Standard chemotherapy	1.00	-	-
	Aggressive-approach chemotherapy	1.32	(0.90, 1.92)	0.15
Second quintile of PS	Standard chemotherapy	1.00	-	-
	Aggressive-approach chemotherapy	1.30	(0.98, 1.72)	0.07
Third quintile of PS	Standard chemotherapy	1.00	-	-
	Aggressive-approach chemotherapy	1.22	(0.81, 1.84)	0.35
Fourth quintile of PS	Standard chemotherapy	1.00	-	-
	Aggressive-approach chemotherapy	1.21	(0.94, 1.56)	0.14
Highest quintile of PS^†^	Standard chemotherapy	1.00	-	-
	Aggressive-approach chemotherapy	0.85	(0.68, 1.07)	0.16
Multiple Cox regression adjustment using the PS	Standard chemotherapy	1.00	-	-
	Aggressive-approach chemotherapy	1.21	(1.00, 1.48)	0.05

Abbreviation: HR, hazard ratio; CI, confidence interval; PS, propensity score.

* Patients are least likely to receive "aggressive-approach chemotherapy within 14 days of death."

† Patients are most likely to receive "aggressive-approach chemotherapy within 14 days of death."

#### 3) Results from instrumental variable analysis

We report selected baseline characteristics of patients according to geographic quintiles of chemotherapy utilization in Table 5. Thirty-one percent of the 1,492 patients in the lowest quintile received chemotherapy, and 52% of the 2,139 patients in the highest quintile received chemotherapy. Patients were similar in most observed characteristics including age, gender, race/ethnicity, and socioeconomic status. In addition, the Cox proportional hazards regression model showed that the IV was not an independent predictor of survival supporting its use as an instrumental variable. Using quintile of chemotherapy utilization as an instrumental variable, we did not observe an association between use of chemotherapy and survival for marginal patients at 1 year (6.7 percentage points; 95%CI -6.6, 19.9%; P = 0.32) or at 2 years (1.7 percentage points; 95%CI -5.9, 9.3%; P = 0.66).

**Table 5:** Instrumental variable analysis results

Model	Patient population	Variable used for classification	Variables	Lowest quintile	Second quintile	Third quintile	Fourth quintile	Highest quintile	Instrumental variable estimate
1	All patients (n = 7,879)	Prevalence of "chemotherapy"	Number of patients*	1,492	1,388	1,439	1,255	2,139	
			Chemotherapy recipients, %^†^	30.8	43.4	47.5	49.8	52.3	
			Aggressive-approach chemotherapy recipients, %^†^	2.2	3.2	4.2	5.1	4.4	
			Age at diagnosis, years	73.0	73.3	72.8	73.3	72.8	
			Female, %	42.2	41.7	42.6	48.3	39.8	
			non-Hispanic white, %	83.7	85.4	88.1	71.9	82.8	
			Socioeconomic status, mean quintile	2.9	2.8	3.0	3.1	3.0	
			Without comorbidity, %	78.2	73.7	74.5	69.8	69.1	
			Survival at 1 year after diagnosis, %^‡^	21.7 ± 1.1	23.5 ± 1.1	22.2 ± 1.1	21.7 ± 1.2	23.7 ± 0.9	6.7% (95%CI: -6.6, 19.9)
			Survival at 2 years after diagnosis, %^‡^	5.6 ± 0.6	6.7 ± 0.7	6.1 ± 0.6	4.8 ± 0.6	6.8 ± 0.5	1.7% (95%CI: -5.9, 9.3)
			Hazard Ratio (95%CI)^§^	1.0	0.94 (0.87, 1.01)	0.96 (0.89, 1.03)	1.04 (0.96, 1.12)	0.93 (0.87, 0.99)	
2	Chemotherapy recipients (n = 3,534)	Prevalence of "aggressive-approach chemotherapy"	Number of patients^||^	453	136	990	619	614	
			Chemotherapy recipients, %	100.0	100.0	100.0	100.0	100.0	
			Aggressive-approach chemotherapy recipients, %^†^	4.6	6.6	7.7	10.3	12.1	
			Age at diagnosis, years	71.7	71.7	71.1	71.7	71.9	
			Female, %	43.7	44.1	38.6	41.7	43.3	
			non-Hispanic white, %	89.0	83.8	84.6	86.0	74.8	
			Socioeconomic status, mean quintile	3.0	3.3	3.2	3.2	3.2	
			Without comorbidity, %	73.5	78.7	70.6	79.2	68.2	
			Survival at 1 year after diagnosis, %^‡^	30.5 ± 2.2	29.4 ± 3.9	26.0 ± 1.4	26.3 ± 1.8	27.9 ± 1.8	-53.5% (95%CI: -124.1, 17.2)
			Survival at 2 years after diagnosis, %^‡^	8.4 ± 1.3	8.1 ± 2.3	7.4 ± 0.8	6.5 ± 1.0	6.2 ± 1.0	-35.1% (95%CI: -76.9, 6.6)
			Hazard Ratio (95%CI)^§^	1.0	1.05 (0.87, 1.28)	1.07 (0.96, 1.20)	1.11 (0.98, 1.25)	1.11 (0.99, 1.26)	

Abbreviation: CI, confidence interval; SE, standard error.

* A total of 166 patients who reside in the Health Care Service Areas, where there were five or fewer patients treated with or without chemotherapy, were excluded.

† P < 0.001

‡ Unadjusted survival derived from Kaplan-Meier method

§ Adjusted hazard ratios and 95% confidence intervals derived from Cox proportional hazards regression model. Patient age, gender, race/ethnicity, socioeconomic status quintiles, Charlson comorbidity index, and year of death were included in the model.

|| A total of 722 patients who reside in the Health Care Service Areas, where there were five or fewer patients treated with or without aggressive-approach chemotherapy, were excluded

Similarly, in model 2, we compared survival across quintiles based on the rate of chemotherapy utilization within 14 days of death among patients who ever received chemotherapy. Five percent of the 453 patients in the lowest quintile received chemotherapy near the end of life, and 12% of the 614 patients in the highest quintile experienced an aggressive approach to chemotherapy. Compared to standard chemotherapy, use of chemotherapy within 14 days of death tended to be associated with worse survival at 1 year (-53.5 percentage points; 95%CI -124.1, 17.2%; P = 0.14) or at 2 years (-35.1 percentage points; 95%CI -76.9, 6.6%; P = 0.10). Because of the relatively small samples of patients receiving chemotherapy near the end of life in geographic quintiles for IV estimates, however, these differences were not statistically significant.

## Discussion

This study explored the clinical effects of chemotherapy continued close to the time of death in a large representative cohort of elderly patients with advanced non-small cell lung cancer in multiple regions of the United States. About one tenth (8.5%) of patients who had ever received chemotherapy were still receiving chemotherapy within 14 days of death; this population experienced no survival benefit as determined by three different statistical approaches. These patients were also much less likely to receive hospice care or and more likely to receive it for three or fewer days. These results suggest that patients receiving chemotherapy within 14 days of death do not benefit from this aggressive approach to treatment, and they may also be deprived of good palliative care provided by hospice.

Results from the IVA confirmed that geographic variations exist in use of chemotherapy, but not in clinical outcomes. This suggests that physician practice styles or patient preferences, rather than associated clinical outcomes, may be driving aggressive treatment decisions near the end of life.

Several limitations should be noted. The SEER-Medicare database is restricted to patients aged 65 or older and patients insured by an HMO were excluded in this study, so it is difficult to extrapolate these results to younger patients and those enrolled in managed care. However, 60% of cancers occur in patients who are 65 years or older,[34] so Medicare covers most cancer patients in the United States [35]. As the data were not collected specifically for research, there may be inaccuracies in some of the variables analyzed, such as the Charlson comorbidity index, but chemotherapy is a costly service with strong financial incentives for accurate billing to Medicare [36,37]. Our data are from patients who died over a decade ago and so treatment options and availability have changed. For example, the modern targeted therapy including an introduction of epidermal growth factor receptor (EGFR) inhibitors [38,39] may have changed the risk/benefit equation. Still, with respect to cytotoxic chemotherapy, our data did not detect any benefit to continuing chemotherapy to near death. Retrospective analyses of patient cohorts defined by death have been criticized because patients' prognosis may often not be apparent to treating clinicians and some deaths may occur despite appropriate efforts to prolong survival [40]. These concerns are mitigated by studying patients who are known to be terminally ill and who die of their terminal illness,[41-43] as we did by focusing on patients initially diagnosed with metastatic non-small cell lung cancer who died of their cancer. A systematic review showed that physicians' survival predictions in terminally ill cancer patients can be inaccurate and generally they are overoptimistic [44]. Palliation is the only goal of therapy for patients with metastatic diseases. In such a case, cure is not an option. If physicians repeatedly give patients chemotherapy very near death because they simply do not see death approaching, they may not be delivering the best care. Similarly, those with a high proportion of unexplained toxic deaths resulted from continuing chemotherapy close to death may be delivering poor care. There may also be a limitation of determining intent of chemotherapy retrospectively: chemotherapy that happens to continue to within 14 days of death may not have been given with the intent of an aggressive approach. However, this is one of the validated measurements that we can use with existing administrative data to assess the intensity of end-of-life cancer care [11,13]. Also, despite the fact that survival time is part of both the dependent and explanatory variables, our data showed that the receipt of aggressive-approach chemotherapy was defined independent of patients' life expectancies, which assuages these limitations. Although observational data do not permit clear determination of causality, the consistency of our findings derived from the three sophisticated statistical approaches with a large sample size is compelling.

## Conclusions

We could not detect a benefit in survival from continuing chemotherapy close to death. Furthermore, this treatment was associated with substantially reduced use of palliative hospice care. The Health Services Research Committee of the American Society of Clinical Oncology (ASCO) agreed that treatment could still be recommended, even without an improvement in survival, if it improves the quality of life in the case of metastatic cancer [45]. Our data suggest that an aggressive approach to continuing chemotherapy to very near death likely does not meet this test in that it may result in no survival benefit, and, in fact, in negative outcomes. It is imperative that physicians present honest, individualized, evidence-based information to patients making treatment decisions near the end of life about the expected risks and benefits of chemotherapy.

## Competing interests

The authors declare that they have no competing interests.

## Authors' contributions

AMS participated in design, conducted the analysis, wrote the manuscript and contributed to the discussion; MBL participated in the design, conducted the analysis, contributed to the discussion and reviewed the manuscript; BAN participated in design, collected research data and reviewed the manuscript; JZA participated in design, contributed to the discussion, and reviewed the manuscript; CCE obtained funding, designed the study, analyzed the data, wrote the manuscript and contributed to the discussion. All authors contributed to data interpretation, made substantive contributions to the manuscript, and had final approval of the article.

## Pre-publication history

The pre-publication history for this paper can be accessed here:

http://www.biomedcentral.com/1472-684X/10/14/prepub
